# University Students’ Achievement of Meaningful Learning through Participation in Thinking Routines

**DOI:** 10.3390/ejihpe14040066

**Published:** 2024-04-11

**Authors:** Nora Ramos-Vallecillo, Víctor Murillo-Ligorred, Raquel Lozano-Blasco

**Affiliations:** 1 Department of Musical, Plastic and Bodily Expression, Area of Didactics of Plastic Expression, University of Zaragoza, 50009 Zaragoza, Spain; vml@unizar.es; 2 Department of Psychology and Sociology, Area of Developmental and Educational Psychology, University of Zaragoza, 50009 Zaragoza, Spain; rlozano@unizar.es

**Keywords:** thinking routine, participation, meaningful learning, academic performance

## Abstract

This study was conducted among undergraduate students pursuing a Primary Education degree at the University of Zaragoza. Its primary aim was to enhance and elevate the quality of participation during theoretical sessions, thereby fostering meaningful learning that contributes to the improvement of academic performance among the participants. To achieve this objective, a quasi-experimental case study was meticulously crafted. This research design was structured to not only stimulate and augment participation but also to cultivate meaningful learning, ultimately enhancing students’ academic achievements. The study employed a comprehensive approach to monitor participation, utilizing observation records to track engagement levels, and anecdotal records to delineate the progression of sessions and the quality of responses. Through meticulous analysis, it can be deduced that the integration of thinking routines as a pedagogical tool in expository sessions significantly enhances student engagement. These routines effectively activate students’ prior knowledge, establishing meaningful connections with the subject matter at hand. Moreover, the incorporation of thinking routines has been observed to elevate the quality of student participation. By fostering reflective practices, aiding in the formulation of inquiries, promoting collaborative learning, and nurturing critical thinking skills, these routines play a pivotal role in enriching the educational experience and bolstering academic performance.

## 1. Introduction

Today’s university students are accustomed to a dynamic and interactive teaching style, and teachers must adjust their practices accordingly. This means integrating curricular and methodological planning that supports active learning and the development of competencies, skills, and abilities that promote communication, collaboration, and engagement with problems. Therefore, it is essential for teacher education programs to give priority to the development of learning experiences that enable active student engagement and foster meaningful relationships within a specific context.

The classroom provides an environment conducive to engaging students in thinking and learning experiences, as well as promoting integration among them [1]. To achieve this, it is important to foster a culture where both individual and group thinking are actively manifested in everyday activities [2]. This educational approach is based on the premise that learning should not only focus on the acquisition of information but also on the development of skills to process and apply that information in meaningful ways. It also recognizes that learning goes beyond the mere accumulation of knowledge, and focuses on the development of skills to analyze, synthesize, and apply information effectively.

Therefore, it is crucial to design innovative curricula that encourage students’ active participation and help them deepen their understanding of fundamental concepts [3]. These curricula should go beyond the transmission of information and actively seek ways to engage students in activities that promote critical thinking, problem-solving, and the practical application of knowledge through the practical use of aesthetic knowledge [4]. In this way, students’ holistic development can be enhanced and they can be prepared to face the challenges of the real world effectively by fostering inquiry and suspicion through the presentation of imaginaries [4].

While active techniques are becoming more prevalent in higher education, it is essential to evaluate their efficacy. Currently, the academic literature does not have sufficient research that thoroughly assesses the efficacy of different thinking strategies and routines in promoting students’ critical thinking and profound comprehension, as well as their influence on the academic achievement of university students. Therefore, this study aims to explore the potential of thinking routines to promote meaningful learning through increased student participation in theory sessions.

Through this study, we seek to fill the gap in the research on the effectiveness of thinking routines as a tool for improving understanding and academic performance at the university level. By analyzing how the implementation of these routines in the classroom can influence student learning, we hope to gain valuable information that can inform future pedagogical practices and educational policies.

### 1.1. Theoretical Framework

Active student participation is a crucial component of the teaching–learning process and represents a central concern for a significant number of teachers [5]. We can distinguish two main approaches in the academic environment: one where students adopt a passive role and another where they take an active role in their learning, known as active learning. In the first, theoretical classes are characterized by the reduced presence of students and the prevalence of the figure of the teacher in what are known as master classes [6]. In contrast, active methodologies involve students in the learning process in a participatory manner, with their not being mere passive recipients of information imparted by the teacher. The basis of active learning is the idea that points to the relevance of the student actively seeking meaning related to the object of their learning, trying to relate it to their previous knowledge, reflecting on its consequences for what they already know, and, in short, thinking about it [7]. It is characterized by stimulating students to generate their own solutions and applying concepts and theories to different situations [8]. Learning with meaning, from what is known, in an active way, and with real tasks, will be the guarantee of authentic learning [8]. Instead of focusing solely on the transmission of knowledge, these methodologies encourage the development of skills such as critical thinking, problem-solving, teamwork, and effective communication, among others [9]. The protagonists of learning are the students.

Another aspect to take into account in this form of learning is that it has teachers engaging in a structured process of analysis and discussion with the goal of generating concrete outcomes. The teacher must teach students to construct mental schemas. Their role, in this type of methodology, is to accompany, guide, give feedback, and evaluate the students during the process. To understand this type of didactic methodology, it is also important to perceive the relevance of student activity in the processes of acquiring cognitive and skill development. New activity-based educational models demand that learners organize their work in a self-sufficient way, which implies a considerable change in the attitude and role of teachers [10]. It seeks to generate more meaningful, relevant and contextualized learning, in which students are the protagonists of their learning process and the teacher acts as a facilitator and guide [11].

Autonomous learning strategies for university students include the students’ contribution to the teaching–learning process, linked to their participation in the classroom [12]. However, it is important to bear in mind that class participation should not only be measured by the number of times a student speaks in class, but also by the quality of their interventions. Teachers should assess whether such participation is conducive to meaningful learning. For this long-term and lasting learning to take place, it is necessary to connect the teacher’s teaching strategy with the students’ previous ideas, presenting the information in a coherent and non-arbitrary way, “building” the concepts in a solid way, interconnecting them with each other in the form of a knowledge network [13].

Thinking culture is an educational approach that fosters the growth of critical and innovative thinking abilities in students, while also encouraging the establishment of a cooperative and introspective learning atmosphere. It promotes the creation of an environment where critical, creative, and reflective thinking is valued, encouraged, and practiced on an ongoing basis. It is based on the idea that learning goes beyond the mere acquisition of knowledge and focuses on the development of thinking skills that enable students to understand, analyze, and apply what they learn in meaningful ways in a variety of contexts. Learning thinking skills develops the deepening and extension of acquired knowledge and stimulates good-quality thinking [14]. Students are encouraged to ask questions, explore different perspectives, express their opinions, and justify their ideas through reasoning. Teachers play a key role in creating and maintaining this culture, using pedagogical strategies that promote reflection, dialogue, and collaboration among students. The aim is to cultivate a mindset of inquiry, analysis, and reflection that prepares students to meet the challenges of today’s world more effectively.

Thus, the boom in the development of a culture of thinking in the classroom has been realized through the design of a series of cognitive tools such as thinking routines, which have contributed to facilitating the acquisition of different types of learning [15,16]. Thinking routines are specific tools to help students develop critical thinking and metacognitive skills. They provide an opportunity to structure and reflect on the processes that take place when we think [17]. These routines provide cognitive structures and processes that guide students through different stages of thinking, such as observation, interpretation, reflection, and synthesis, thus facilitating deeper and more meaningful thinking.

They are presented as a very useful pedagogical resource for generating ideas, enhancing reasoning skills, encouraging reflection, and understanding school content more effectively [18]. In addition, they make the knowledge, ideas, reflections, and hypotheses that students carry out visible in that they favor the creation of reflective mental habits, the generation of ideas, the organization of mental actions, and the establishment of relationships, as well as the coherent structuring of content, something that will favor the future employability levels of a person in the productive fabrics. This is the way they are because, as Aguirre [19] points out, education aims to create free and creative subjects, something that modern society demands for any of its spheres.

### 1.2. Objective and Research Questions

This study aims to analyze the differential value of the teaching methodology of thinking routines, used in the subject of “Visual and plastic education” in the degree program for becoming a teacher in Primary Education, as a didactic tool that favors participation in the classroom and increases academic success.

Based on this objective, we posed the following research questions:

Q1: Is there greater student participation in the sessions carried out using thinking routines?

Q2: To what extent does the teaching methodology of thinking routines contribute to students’ meaningful learning and improve their academic performance in aspects such as the final grade of the subject, their exam grade, practical grade, and project grade?

Q3: Are there significant differences in students’ academic performance and participation according to variables such as gender and age?

## 2. Methodology

### 2.1. Sample

We present a convenience sample of N = 237 university students aged between 20 and 49 years, with a mean age of 20.90 years (SD = 3.46). Of these, 170 are female and 67 are male. They are divided into four teaching groups, the first two participating in the morning and the second two in the afternoon.

### 2.2. Teaching Approach

The subject in which the study was carried out is “Visual and plastic expression”, a compulsory subject in the second year of the Primary Education degree, with 6 ECTS credits.

Classes are structured in two sessions per week for each group, each lasting 110 min:

- In the single-group theory sessions, where only one teacher intervenes, they correspond to the explanation of the theoretical concepts.
- In the practical sessions, work is carried out by splitting the groups, with one teacher in each group. Due to the characteristics of the problem-solving and case-solving part of the course, eminently practical issues are dealt with.

The final grade for the course is made up of individual practicals, group projects, and a theory test (see Table 1).

The arrangement of the theoretical topics and their structure when they are taught are designed to have a direct relationship with the individual practices and group projects carried out in parallel with the practical sessions. Thus, the connection between theory and practice is perfectly interwoven to anticipate practical processes with theoretical knowledge.

The theoretical part of the course consists of a total of 9 sessions, 4 of which are structured through the methodology of thinking routines and the other 5 follow a traditional expository methodology.

In the sessions where the thinking routines were implemented, we tried to maintain the structure of the sessions. In each work session (four in total), three routines were developed: one at the beginning of the class to present and explore ideas; another after explaining the general presentation of the topic to synthesize the information; and the last at the end of the session to organize and deepen the concepts presented [20].

The thinking routines that were selected were more in line with the development of the conceptual content of the subject, each with a different objective (see Table 2).

Different methods were used to measure a student’s class participation:

- Direct observation: The teacher observed and recorded in all theory sessions the frequency and quality of the students’ interventions in the class. This was recorded in a checklist.
- Questions and answers: The teacher asked students questions during the theory classes where thinking routines were not used, and evaluated their answers. This allowed the level of understanding and participation of the students to be assessed.

### 2.3. Design and Procedure

This is a quasi-experimental research study since, due to the characteristics of the university classroom, it is not possible to apply random or stratified sampling, leading us to opt for a convenience sample. Thus, a case study (groups) is applied in which the teachers play a mixed researcher–teacher role. This research design aims to respond to two real classroom needs: (a) to encourage and increase participation and (b) to promote meaningful learning that improves students’ academic performance. In this way, participation was recorded using an observation register, and the quality of the responses was recorded using an anecdotal record in which the teacher explained the development of his or her sessions.

**Figure 1:**
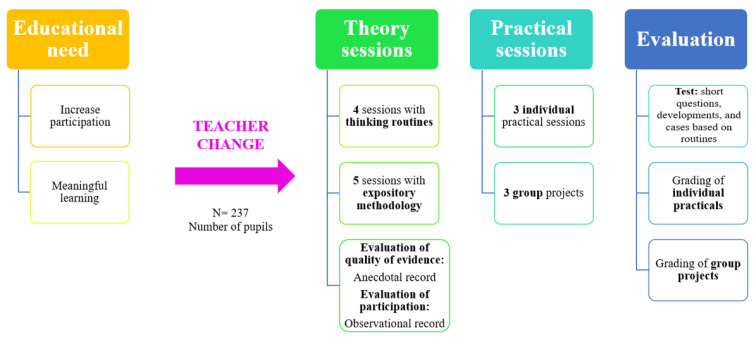
The study’s design and procedure. Source: own elaboration.

The procedure followed and previously described in Section 4.2 is shown in the following figure (see Figure 1).

### 2.4. Variables and Instruments

The following study variables were found:

Learning routines: teaching tools used to develop critical thinking skills and promote a reflective approach to problem-solving [17].

Expository methodology: an educational approach in which the teacher presents information in a direct and organized manner to students. It is a form of teaching in which the teacher conveys knowledge, concepts, or skills through verbal exposition, often supported by visual materials such as slide presentations, whiteboards, or multimedia resources [21].

Academic performance: refers to the level of success a student achieves in their educational activities, in this case, measured by continuous assessment work and an end-of-course exam [22].

Socio-demographics: gender (male, female) and age (year of birth) are considered.

In addition, the following methods were used as instruments to collect information:

Anecdotal recording: this is used to assess the quality of the evidence. It is a qualitative observation technique in which detailed and objective descriptions of specific student behaviors in the classroom context are collected [22,23]. To ensure the reliability and usefulness of the anecdotal record as a measurement tool, the following principles were followed [22,23].

- Objective observation (based on observable facts, avoiding the inclusion of personal judgments or subjective interpretations).
- A clear context (including details about the classroom, the activity, or any other relevant factor that may influence the interpretation of the observation).
- Specificity (specific behaviors, avoiding generalizations).
- Consistency (being consistent with the observation approach and criteria so that notes are comparable over time and between different observers).
- Immediacy (annotations are made as soon as possible to ensure accuracy and avoid losing important details).
- Periodic recording (records are made at each session, i.e., regularly, to assess the progress of and changes in the subject throughout the study).
- Confidentiality and ethics (the anonymization and privacy of data being kept and following the ethical principles set out in the Helsinki Declaration) [24].

Observational recording: used to assess student participation. It is a data collection technique that involves the systematic and recorded observation of behaviors in the natural classroom environment [22,23]. To maintain the reliability and internal consistency of the instrument, the following principles were followed:

- The clear definition of behaviors (student participation, considering its quality and frequency).
- The training of observers and inter-observer reliability (observers are experienced teachers who have been teaching the subjects in a coordinated manner for more than 4 years).
- The standardization of procedures (being held in the plastic education classroom, during practical and theoretical sessions throughout the whole subject).
- The use of clear categories or codes (tips are used to indicate the number of times students participate, and written annotations are used to describe the quality of the intervention).
- Real-time recording (while the session is taking place the teacher who is a participant observer keeps a record of everything).
- Contextual recording (recording information about the classroom context)
- Periodicity and duration (carried out during all theoretical and practical sessions throughout the semester) [22,23].

## 3. Results

### 3.1. Results regarding Question 1: Is There Greater Student Participation in the Sessions Carried out Using Thinking Routines?

Initially, the spontaneous participation of the students is practically nil during the sessions with expository methodologies, whereas, if the teaching methodology of thinking routines is used, the students increase their spontaneous participation (see Table 3). In this sense, 39.24% of the students participated in the sessions with routines. This affirmatively answers the first research question, Q1.

Furthermore, it is necessary to study the value of the socio-demographic variables of the students. The ANOVA test found no differences between groups, while the Student’s *t*-test did show significant differences between sexes, with males participating more. Finally, the ANOVA test did not show significant differences between age groups (ANOVA; F = 2.79, *p* = 0.096) (see Table 3).

### 3.2. Results regarding Question 2: To What Extent Does the Teaching Methodology of Thinking Routines Contribute to Students’ Meaningful Learning and Improve Their Academic Performance in Aspects Such as the Final Grade of the Subject, Their Exam Grade, Practical Grade, and Project Grade?

Consequently, it is necessary to verify, in the first instance, whether there are significant differences between the students who participate regularly in the routine sessions and the academic success variables by employing an ANOVA test (see Table 4). Thus, significant differences are found in favor of those who do participate in the assessments for the class practicals and projects, i.e., in the continuous assessment sections. The methodological change does not improve the final exam grade, or that of the subject.

Regarding the quality of the participation in the thinking routines sessions and the academic success variables, a series of simple forward stepwise regressions are performed (see Table 5). In this way, the effect of the quality of students’ responses in the routine sessions on each of the academic success variables is analyzed. In summary, it is found that 2% of the final subject grade, 3% of the practical grades, and 2% of the final project grade are significantly explained by the quality of the student’s answers in the routines (see Table 5). However, the routine methodology does not explain the final exam grade in percentage terms. Consequently, research question Q2 is partially affirmatively answered.

On the other hand, it is necessary to expose how the Student’s *t*-test shows no differences between sexes (F = 2.13; *p* = 0.14; t = −1.85; *p* = 0.06) except for their participation, where men stand out. However, the age variable is relevant. A simple regression analysis shows that 3.5% (R^2^ = 0.035) of the quality of the response is mediated by age (t = 2.92; *p* = 0.004), showing a positive relationship. In other words, as age increases, the quality of responses increases. Thus, Q3 is partially affirmatively answered given that, although there is no difference between the quality of the ratings, men participate to a greater extent than women, and that the quality of the responses in routines is mediated by age.

### 3.3. Results regarding Question 3: Are There Significant Differences in Students’ Academic Performance and Participation According to Variables Such as Gender and Age?

It is necessary to test the holistic relationship between the methodology of routines and the academic success variables together with the moderating variables in such a way that a comprehensive model is obtained by using moderation analysis [25] (see Table 6).

This model reaffirms the results found, as there is a direct effect of the quality of participation in routines on the final grades for the subject, the practical and project grades (see Table 7).

Furthermore, this allows us to rule out age as a moderating variable through its indirect effects (see Table 8). In other words, in the overall analysis of the subjects’ evaluations, age is not significant.

However, the residual effects (see Table 9) show how the grades for the project and the practical exercises have a very strong and positive effect on the final exam grade. In short, improving the performance in internships and projects increases the excellence in the objective assessment of the examination. In other words, continuous assessment leads to better learning.

Gender is a determining factor in the quality of the contributions in the routine sessions and the continuous assessment grades. Thus, the female gender plays a certain role in the practical (–0.56) and project (–0.86) grades, but not in the final and exam grades, where the values are very low. Consequently, the reason for these differences should be further explored, as the evolutionary stage of university students does not justify such differences. Finally, it should be noted that research question Q3 is partially affirmatively answered, since gender plays a determining role while age shows very weak values.

## 4. Discussion

This study analyses student participation from a pedagogical perspective, considers the characteristics of the participants, and is conceived as a tool for developing attitudes, regulating procedures, and learning strategies [25]. Different research reveals how the learning strategies of university students are related to their academic results [26,27], and this link is associated with individual variables such as age, gender, and learning style [28,29,30]. All of this establishes a relationship between learning approaches and the self-regulation that students develop, concretized in better planning and control of execution [31,32], favoring deeper learning, and observed in greater planning and behavior through self-regulatory strategies.

Thinking routines are shown to encourage engagement in learner interventions. This is an effective tool for learning, given that knowledge is acquired through the involvement of student activity [33,34,35,36,37]. We can affirm that it is more complex to encourage participation in a classroom where theoretical concepts are developed, and the teacher controls the speaking time to a greater extent [38].

It is important to remember that participation is not innate and is learned through practice, which makes it even more important to try to educate with it [39]. Considering the good of Dewey’s principle [40] of learning-by-doing, there is also doing-by-learning. Among the main findings, in this respect, is the foundation of active methodologies in constructivist theory. These focus the teaching–learning process of the students, as well as their uniqueness in favoring active participation and cooperative working relationships, having the resolution of real problems as a didactic-methodological resource, rejecting the rote process, and pursuing creativity and critical reflection [6]. This undoubtedly requires changes in the forms of planning with the design of didactic activities that promote student participation and evaluation in such a way that the learning process responds to a constructivist perspective [41].

From this conviction, the didactic strategies available to teachers which are valuable tools for transforming teaching and the teaching–learning process aim to put the student at the center of the process, where teaching does not revolve around the teacher and the content, but rather around the student and the activities they carry out to achieve learning [41]. In this sense, we have found that active methodologies lead to greater student participation and interest. These didactic methods have some characteristic features such as promoting the construction of meaningful learning, bringing specific student skills into play, and stimulating critical thinking and creativity to make predictions [42]. Thinking about the training process from these active methodologies does not mean incorporating isolated activities that promote participation, but rather it implies thinking about teaching at the service of the student [41], with these strategies, such as thinking routines, being an ideal ally for the promotion of critical thinking and reflection through iconic discourses.

However, it is important to develop theoretical content oriented to the application of the necessary knowledge for the solving of practical problems [9]. Therefore, from the experiences generated by thinking routines, it is possible to stimulate students and, at the same time, give value to what they discover through their interpretations [42]. A student’s participation in class is a didactic strategy for learning from challenges, making it an enjoyable learning practice and fostering many interpersonal skills and abilities [43]. In our results, these skills and abilities are associated with an improvement in the quality of practices and projects. Students acquire a higher level of commitment to what they have studied, favoring autonomy, and generating competencies for learning to learn in collaboration with peers [41].

For example, for future professional student teachers, it is important to increase their communication skills, but these are skills that are not usually given enough attention and are always addressed transversally [44]. Using thinking routines, participation is stimulated with the use of questions and reflections in an oral form, leading to the development of effective communication strategies. These allow students to develop the higher-order skills, such as collaboration and self-learning, that are demanded by society and are useful not only in their academic life but also in their professional life [45].

Moreover, since reflection is carried out in groups, students are allowed to share, contrast, and discuss their ideas with their classmates, as well as with teachers, in a relaxed atmosphere, with very positive effects on learning [7]. By using thinking strategies and routines, the culture of thinking in the classroom encourages reflection and the analysis of concepts. Students are challenged to think deeply about concepts, consider different perspectives, and evaluate evidence before reaching conclusions.

Through the implementation of thinking routines in a university classroom, we have observed an enhancement in the cultivation of consideration and respect for a diversity of opinions. By creating an environment where the expression of different points of view is valued and respected, the thinking culture in the classroom promotes an openness to diversity and a respect for the opinions of others. A culture of thinking encourages students to consider and explore different perspectives on a given topic. Through discussion and debate, they are challenged to critically examine their own beliefs and consider new ideas. Exposure to different points of view enriches learning by providing students with a more complete and nuanced understanding of an issue. It helps them develop flexible thinking skills and appreciate the complexity of issues from multiple perspectives.

Exposing students to different points of view and encouraging them to consider diverse opinions fosters critical thinking and enriches learning, which is an original contribution to students’ development as global citizens and independent thinkers. Contributions related to the culture of thinking in the classroom are effective because they focus on the development of thinking skills, promote an environment of inquiry and exploration, use innovative thinking strategies and routines, and value diversity in perspectives as an integral part of the learning process.

In the present study, participation was analyzed considering the promotion of learning situations involving subject concepts to promote the construction of significant learning from a theory test, practicals, and projects [46].

Regarding the academic performance demonstrated in the results of the theory test, we cannot confirm that the use of routines was effective, unlike in the cases of the practicals and projects. As in other studies [47] where tools to encourage participation, such as digital questionnaires designed with SOCRATIVE, were used, no improvement in academic results was observed since the concepts had not been adequately assimilated.

The effectiveness of the methodological proposal used in this study corresponds to the model designed at Harvard University [5], where the content developed in the projects is learned using a methodology based on learning and not so much on teaching. In other words, the teaching work is present in the design of the routines, their monitoring, and their sharing so that the content that the students develop practically in the projects is learned through participatory and reflective strategies. The work was based on a process guided by the teachers to promote spaces for autonomous work, the social construction of knowledge, and critical participation, thus favoring the desired cognitive, procedural, and attitudinal development of the students [48].

The students who participated in the study, during the internships and projects, worked actively and contributed to the group work in a representative way, which can be considered to be participation in class. The thinking culture encourages the development of critical thinking skills, such as analyzing, evaluating, and synthesizing information. These skills are essential for students to understand and question the world around them in a deeper and more meaningful way. The thinking culture focuses not only on understanding concepts but also on applying them in practical situations. Encouraging students to think critically about how they can use concepts in real life helps them to develop knowledge-transfer skills.

We can conclude that, if we want to encourage participation in classrooms to improve academic performance and achieve meaningful learning, the choices will range between teacher-centered and student-centered teaching methods. Between these two methodological assumptions, it is possible to establish a continuum of combinations with differential participation being one of the extremes. Concerning learning, the choice would fluctuate between favoring rote, reproductive, and superficial learning, or meaningful learning through understanding, investigation, and in-depth knowledge [43]. We have been able to confirm that spending time in theoretical classes to mobilize students’ prior knowledge promotes the integration of new knowledge effectively into the practical part of the subject [49].

Through this study, we have found a correlation between encouraging the development of tools that foster a culture of thinking in university classrooms and promoting a focus on deeply understanding concepts rather than simply memorizing information. By encouraging students to think critically about concepts, you help them better understand the concepts’ meaning and applications.

A culture of thinking in the classroom can lead to better academic performance by students. By promoting more active and reflective thinking, students can improve their understanding of concepts and apply them more effectively in various academic situations. Furthermore, in an increasingly complex and changing world, critical, creative, and reflective thinking skills are increasingly important. The culture of thinking in the classroom helps prepare students to face real-world challenges by fostering the ability to solve problems, make informed decisions, and communicate effectively. By promoting an environment where active and reflective thinking are valued and encouraged, a classroom’s thinking culture can increase students’ participation and commitment to the learning process. Students feel more motivated and engaged when they are allowed to think critically and actively contribute to classroom discussions.

It can be stated that teaching methods with student participation, where the responsibility for learning depends directly on students’ activity, involvement, and commitment, are more formative than merely informative, generate deeper, more significant, lasting learning, and facilitate the transfer of knowledge to more contexts heterogeneously [43].

The present study is in line with previous research where socio-demographic variables have some moderate power. Regarding the relevance that the students’ genders may have on their participation, there are many studies on the subject that describe a broad panorama of their differences in the use of learning strategies and styles [28]. Focusing on the Spanish territory and the university students of a Primary Education degree, in a teaching innovation experience of the faculty of education of the Complutense University of Madrid [50], they have identified that men, despite being fewer in number, tend to intervene in all classes. This same report highlights that the participation of female students is lower, although their contributions are more argumentative. The present study coincides with studies where no significant differences were found in terms of the quality of participation [44].

There is little theoretical foundation to justify the age differences in the study processes of higher education students. It has been found in this research that older students develop higher-quality strategies than younger students do through their participation in thinking routines. These results are in line with the findings of Richardson [51], whose research has shown that older students tend to use a deeper approach, while younger students adopt a more superficial approach. In contrast, other authors [52] consider that the mere fact that students in higher education reach a certain age does not mean that they have mature learning strategies. Considering the above, it can be justified that older university students have a higher level of personal and/or professional responsibility, which results in greater class participation, which leads them to develop their university studies more successfully due to their degree of involvement and performance in them.

Beyond the age and sex of the students, we consider that participation improves coexistence and the transformation of educational practices towards democratic models and equity [25].

In short, the mere participation in the sessions with thinking routines means that the students who have participated in the study earned better grades on the practicals and in the projects of the subject, that is, in the sections of continuous assessment.

### 4.1. Limitations and Prospective Studies

This study is subject to some limitations. Specifically, since it is a quasi-experimental study involving only 237 students from the Education department at the University of Zaragoza, it may be difficult to generalize the findings to other samples. This raises several important questions that demand further investigation: Are there differences in participation rates between students from different university degrees or branches of knowledge? Do students from private and public universities behave differently? How do intrinsic and extrinsic motivation impact student participation? Is the teaching vocation a critical factor? What role do socio-demographic variables play?

To address these issues and to expand the scope of this study, we plan to broaden the sample to include students from other fields and disciplines. We also plan to compare the adoptions of these strategies across students enrolled in different universities (public and private). Additionally, we will examine the impact of motivation and the teaching vocation and explore in greater depth the significance of socio-demographic variables such as gender and age. The current scientific evidence suggests discrepancies that require further analysis.

### 4.2. Practical Applications

Due to the limited knowledge base in the early stages of higher education, incorporating thinking routines can enhance active participation and reflection during practical sessions. These routines can also be useful for subjects that rely heavily on memorization. To fully benefit from these routines, a strong understanding of the subject matter is necessary. Thinking routines provide invaluable tools to comprehend theoretical concepts across various fields of knowledge, not just in university courses, but also in secondary education. By encouraging participation and promoting meaningful learning, thinking routines can offer benefits to students in all subjects.

## 5. Conclusions

Through this work, we can conduct a preliminary analysis of the efficacy of thinking routines in promoting student engagement and facilitating meaningful learning among university students. This insightful research brings university educators one step closer to comprehending the factors that encourage students to participate actively in class, thanks to the implementation of this effective pedagogical tool that enhances training activities.

The findings of this study validate the value of learning routines in substantially enhancing both teaching and learning processes:

1. They develop reflection: Thinking routines encourage students to reflect on their learning, enabling them to think critically about the concepts they are being taught. This can improve their ability to participate in the classroom because they are more engaged with the content and have a better understanding of it.
2. They help to formulate questions: Thinking routines help students formulate questions about content, allowing them to participate more actively in the classroom. By asking questions, students can deepen their understanding and get answers to their concerns.
3. They promote collaboration: Thinking routines drive collaboration among students, which enhances student engagement. When students work together to apply a thinking routine, they can discuss their ideas and build knowledge together.
4. They increase critical thinking skills: Thinking routines are designed to develop critical thinking and metacognition skills in students. When students use these skills, they are better equipped to actively engage in learning and question what they are being taught.
5. They encourage the exploration of multiple perspectives and solutions to complex problems and encourage students to question assumptions and consider the consequences of their decisions and actions. They also emphasize the importance of effective communication, active listening, and collaboration in learning.

Routines increase spontaneous participation in the classroom. They are a good strategy to actively involve students by motivating them using images and their verbalization. The aim is to get students out of passivity and to make them aware of the process of the self-regulation of their learning. We consider that through this didactic tool, they can develop thinking habits that improve the quality of their participation in the classroom and they can apply them to practical problem-solving situations. In terms of socio-demographic factors, our findings indicate that there are significant differences in participation based on gender and age. Specifically, men tend to be more participative than women, and older students tend to be more active than younger ones.

Furthermore, even though we did not observe any individual exam success, we found that students who participated in sessions through learning routines showed greater academic success, particularly in their individual practicals and group projects. This highlights the significant impact of using routines in theoretical classrooms, leading to better practical academic performance.

Through the space for reflection that is established by learning routines, students develop a deeper understanding and sense of project work. This has led to improved creativity, a better comprehension of objectives, and a greater involvement in proposals.

## Figures and Tables

**Table 1:** The final evaluation of the course is made up of practicals, projects, and a theoretical test.

Practical	Projects	Teoretical Test
Practical 1. 10%	Projects 1. 15%	To be taken at the end of the teaching period of the four months, within the class sessions’ data.
Practical 2. 10%	Projects 2. 15%	
Practical 3. 15%	Projects 3. 15%	
		Total, Theoretical test: 20%
Total:		100% of the subject

Source: own elaboration.

**Table 2:** The thinking routines used in the subject.

Thinking Routines Employed	Objective	Thinking Routines Employed
I see, I think, I wonder	To look for the essential questions	Identification of significant questions related to a topic. Consideration of different perspectives and viewpoints on a topic.
Viewpoints	Think in perspective	
Compare and contrast	Make connections	Identifying relationships and connections between different ideas, concepts, and issues.
Focus	Search for evidence	Search for relevant evidence and data to support an opinion or argument.
Headline	Summarize and synthesize	Ability to summarize and synthesize complex information in a simpler and more understandable format.
Ten times two	Observing and describing	Observation and detailed description of an object or situation.
Beginning-middle-end	Thinking about consequences	Consideration of the possible consequences of an action or decision.

Source: own elaboration.

**Table 3:** The average participation of students in the sessions with the thinking routines methodology. Source: own elaboration.

Average Participation in Sessions with Routines				
		They Do Not Use Routines Frequently	They Do Use Routines Frequently	Total
Groups	1	36	27	63
	2	40	24	64
	3	33	21	54
	4	35	21	56
	Total	144	93	237
ANOVA Levene = 2.8; *p* = 0.05 ANOVA; F = 0.16, *p* = 0.92				
Gender	0 female	112	58	170
	1 male	32	35	67
Student’s *t*-Test Levene = 2.13; *p* = 0.01 ANOVA; F = 7.10, t = −2.53; *p* = 0.008 Mean female = 0.53, DS = 0.47; Mean male = 0.52; DS = 0.50				
Age	Young adult 20–29	143	89	232
	Average adult 30–49	1	4	5
ANOVA Test Levene = 8.12; *p* = 0.005 ANOVA; F = 2.79, *p* = 0.096				

Source: own elaboration.

**Table 4:** ANOVA analysis between the students who do or do not participate in the routine sessions and the academic success variables.

		N	Mean	Dev. Diversion	F	Sig
FINAL GRADE	Do not participate	137	7.95	0.92	2.99	0.08
	Do Participate	93	8.16	0.86		
	Total	230	8.03	0.90		
FINAL EXAM GRADE	Do not participate	136	338.64	3865.30	0.85	0.35
	Do participate	93	978.69	6587.91		
	Total	229	598.57	5143.75		
PRACTICAL GRADE	Do not participate	144	7.73	2.12	6.93	0.009
	Do participate	93	8.33	0.78		
	Total	237	7.97	1.74		
PROJECT GRADE	Do not participate	144	7.81	2.19	8.83	0.003
	Do participate	93	8.52	0.91		
	Total	237	8.09	1.83		

Source: own elaboration.

**Table 5:** Simple regression coefficients of the quality of participation in thinking routines, the final subject grade, the final exam grade, and the practical and project grades.

Model		Unstandardized Ratios		Standardized Ratios	t	Sig.	R^2^
		B	Desv. Error	Beta			
1	Final Grade						
	(Constant)	7.95	0.06		116.00	<0.00	
	Student Participation in Routines	0.01	0.005	0.15	2.35	0.02	0.02
2	Final Exam Grade						
	(Constant)	460.95	396.15		1.16	0.24	
	Student Participation in Routines	18.45	27.19	0.04	0.46	0.64	<0.00
3	Practical Grade						
	(Constant)	7.78	0.12		60.21	0.000	
	Student Participation in Routines	0.02	0.009	0.18	2.18	0.005	0.03
4	Project Grade						
	(Constant)	7.92	0.13		58.15	<0.00	
	Student Participation in Routines	0.02	0.01	0.15	2.44	0.01	0.02

Source: own elaboration.

**Table 6:** Total effects.

Total Effects								
							95% Confidence Interval	
			Estimate	Std. Error	z-Value	*p*	Lower	Upper
Student Participation in Routines	→	PRACTICAL GRADE	0.015	0.005	3.264	0.001	0.006	0.024
Student Participation in Routines	→	PROJECT GRADE	0.014	0.005	2.764	0.006	0.004	0.024
Student Participation in Routines	→	FINAL GRADE	0.014	0.004	3.303	<0.001	0.005	0.022
Student Participation in Routines	→	FINAL EXAM GRADE	0.006	0.008	0.814	0.416	−0.009	0.021

Delta method standard errors, normal theory confidence intervals, ML estimator. Source: own elaboration.

**Table 7:** Direct effects.

Direct Effects								
							95% Confidence Interval	
			Estimate	Std. Error	z-Value	*p*	Lower	Upper
Student Participation in Routines	→	PRACTICAL GRADE	0.015	0.005	3.191	0.001	0.006	0.024
Student Participation in Routines	→	PROJECT GRADE	0.013	0.005	2.575	0.010	0.003	0.024
Student Participation in Routines	→	FINAL GRADE	0.012	0.004	2.967	0.003	0.004	0.020
Student Participation in Routines	→	FINAL EXAM GRADE	0.005	0.008	0.679	0.497	−0.010	0.020

Delta method standard errors, normal theory confidence intervals, ML estimator. Source: own elaboration.

**Table 8:** Indirect effects.

Indirect Effects										
									95% Confidence Interval	
					Estimate	Std. Error	z-Value	*p*	Lower	Upper
Student Participation in Routines	→	AGE	→	PRACTICAL GRADE	3.019 × 10^−5^	9.502 × 10^−4^	0.032	0.975	−0.002	0.002
Student Participation in Routines	→	AGE	→	PROJECT GRADE	7.083 × 10^−4^	0.001	0.658	0.511	−0.001	0.003
Student Participation in Routines	→	AGE	→	FINAL GRADE	0.001	9.180 × 10^−4^	1.280	0.201	−6.245 × 10^−4^	0.003
Student Participation in Routines	→	AGE	→	FINAL EXAM GRADE	9.130 × 10^−4^	0.002	0.581	0.561	−0.002	0.004

Delta method standard errors, normal theory confidence intervals, ML estimator. Source: own elaboration.

**Table 9:** Residual covariances.

							95% Confidence Interval	
			Estimate	Std. Error	z-Value	*p*	Lower	Upper
PRACTICAL GRADE	↔	PROJECT GRADE	0.469	0.063	7.412	<0.001	0.345	0.593
PRACTICAL GRADE	↔	FINAL GRADE	0.395	0.051	7.735	<0.001	0.295	0.495
PROJECT GRADE	↔	FINAL GRADE	0.470	0.058	8.153	<0.001	0.357	0.583
PRACTICAL GRADE	↔	FINAL EXAM GRADE	0.208	0.082	2.536	0.011	0.047	0.369
PROJECT GRADE	↔	FINAL EXAM GRADE	0.279	0.092	3.047	0.002	0.099	0.458
FINAL GRADE	↔	FINAL EXAM GRADE	0.691	0.085	8.164	<0.001	0.525	0.857

Delta method standard errors, normal theory confidence intervals, ML estimator. Source: own elaboration.

## Data Availability

Data are contained within the article.
